# Analysis of the clinicopathological characteristics and prognosis of triple-positive breast cancer and HER2-positive breast cancer—A retrospective study

**DOI:** 10.3389/fonc.2022.999894

**Published:** 2023-01-12

**Authors:** Dongxu Ma, Qing Yang, Ke Yin, Peng Shi, Xiao Chen, Tianyi Dong, Xingchen Shang, Xingsong Tian

**Affiliations:** ^1^ Department of Breast and Thyroid surgery, Shandong Provincial Hospital, Shandong University, Jinan, Shandong, China; ^2^ Department of Breast and Thyroid Surgery, Shandong Provincial Hospital, Shandong First Medical University, Jinan, Shandong, China; ^3^ Department of Pathology, Shandong Provincial Hospital, Shandong University, Jinan, Shandong, China

**Keywords:** triple-positive breast cancer, HER2-positive breast cancer, clinicopathological characteristics, prognosis, hormone receptors, adjuvant chemotherapy, targeted therapy

## Abstract

**Background:**

Adjuvant chemotherapy and targeted therapy have become standard postoperative therapeutic modalities for human epidermal growth factor receptor 2 (HER2)-positive breast cancer(HER2-positive,HR-negative), including triple-positive breast cancer(HER2-positive,HR-positive). However, these two types of breast cancer differ in terms of pathogenesis. This article analyzes these two types of breast cancer by comparing their prognoses.

**Methods:**

The clinicopathological characteristics of 135 patients, including 60 patients with triple-positive breast cancer and 75 patients with HER2-positive breast cancer, were analyzed to compare the disease-free survival (DFS) and overall survival (OS) of the two groups over a 5-year period. A multifactorial Cox risk model was constructed by grouping age, menstrual status, maximum tumor diameter, number of lymph node metastases, pathological staging, and Ki-67 staining results. All statistical data were analyzed in detail using SPSS25.0 statistical software.

**Results:**

The 5-year OS rates of patients with breast cancer in the triple-positive and HER2-positive groups were 96.7% and 82.7%, respectively, and the 5-year DFS rates were 90% and 73.3%, respectively. The Cox results revealed that molecular staging was an independent factor affecting recurrent metastasis and survival of breast cancer patients (hazard ratio [HR] =2.199, 95% confidence interval [CI], 1.296-8.266; HR = 9.994, 95% CI, 2.019-49.465).

**Conclusion:**

The 5-year DFS and OS rates were significantly better in the triple-positive group than in the HER2-positive group. Subgroups received different prognosis for different chemotherapy regimens. Breast cancer patients should be treated according to the risk of recurrence with symptomatic treatment and precise regulation.

## 1 Introduction

Breast cancer is one of the most common malignant tumors in the world, and its incidence has been increasing every year (1). In 2020, the World Health Organization’s International Agency for Research on Cancer released the latest global cancer burden data for 2020, which showed that the global number of new breast cancer cases reached 2.26 million, exceeding the second-placed lung cancer by 60,000 cases and accounting for 24.5% of the total number of new cancer cases in women worldwide; the disease was also responsible for 680,000 deaths, ranking fifth in the world (2). In February 2022, the China National Cancer Center released the latest edition of national cancer statistics(This report collects a summary of the information registered in 2016), with 306,000 new cases of breast cancer and 72,000 deaths. Breast cancer incidence and mortality rates are on the rise in China (3). Human epidermal growth factor receptor 2 (HER2)-positive breast cancer accounts for 20%-30% of all breast cancers, with HER2-positive/hormone receptor (HR)-positive cases accounting for approximately 50% of the cases and HR-negative cases accounting for approximately 30% (4). Both from the epidemiological point of view and later drug development, HR-negative/HER2-positive and HR-positive/HER2-positive breast cancers show substantial differences in terms of treatment modalities. With the improved understanding of the biological behavior of breast cancer and the gradual updating of treatment concepts from simple surgical treatment to chemotherapy, combined radiotherapy, and combined targeted therapies, as well as the constant improvements in targeted drugs, the treatment of breast cancer has stepped into a new era (5). The 2021 NCCN Breast Cancer Guidelines (6) state that adjuvant chemotherapy and targeted HER2 therapy are required for both categories of breast cancer after surgery, with the exception of HR-positive breast cancers, for which endocrine therapy is an additional option. The 2021 CSCO breast cancer guidelines (7) also recommended adjuvant chemotherapy combined with targeted therapy; thus, adjuvant chemotherapy and targeted therapy have become the standard postoperative therapeutic regimens for HER2-positive breast cancer, including triple-positive breast cancer. For some patients with intermediate to advanced disease, neoadjuvant chemotherapy can also be the initial treatment option, Highly recommended neoadjuvant treatment options for both types of breast cancer are paclitaxel + carboplatin + trastuzumab+patuximab (TCbHP)and paclitaxel + trastuzumab+patuximab (THP).There are many options for postoperative adjuvant therapy. Trastuzumab combined with paclitaxel chemotherapy can be the basic treatment option for HER2-positive breast cancer. However, the pathogenesis of these two types of breast cancer is different, and the interaction between the HR and HER2 signaling pathways in triple-positive breast cancer (8) may interfere with treatment. Thus, the two types of breast cancer treatment options require different treatment options. In this paper, these two types of breast cancer were analyzed by comparing their prognoses.

## 2 Methods

The clinicopathological characteristics of 807 breast cancer patients admitted to our hospital from January 2014 to December 2015 were evaluated to identify 150 breast cancer patients who met the enrollment evaluation criteria and were followed up until December 2020; 15 patients were lost to follow-up, representing a loss rate of 10%. The inclusion criteria were as follows: (1) Patients were all female. (2) Patients were all first-time patients. (3) The diagnosis of breast cancer was confirmed by pathological histological examination. (4) The clinical data of the patients were complete. (5) Patients underwent modified radical breast cancer surgery and postoperative adjuvant treatment. (6) The follow-up data of the patients were complete. The exclusion criteria are as follows: (1) The patient is male. (2) Both sides have breast cancer. (3) Patients with occult breast cancer. (4) Patients with Ductal Carcinoma *In Situ* (DCIS). (5) Preoperative neoadjuvant chemotherapy. (6) Patients with incomplete basic case information. (7) Patients with malignant tumors of other tissues and organs in addition to breast cancer. (8) Stage IV breast cancer. (9) Patients with no follow-up after treatment or no follow-up information. The data of the remaining 135 patients, including 60 patients with triple-positive breast cancer and 75 patients with HER2-positive breast cancer, were collected, and their clinicopathological characteristics, including age, menstrual status, maximum tumor diameter, number of lymph node metastases, pathological stage, estrogen receptor (ER), progesterone receptor (PR), and Ki-67 statuses, chemotherapy regimen, and postoperative combination therapy, were evaluated. The electronic medical records were used to query the pathology database, and telephone follow-up assessments were used to capture the prognosis and survival of the patients. Chemotherapy regimen choices included adriamycin + cyclophosphamide sequential paclitaxel + trastuzumab (AC-TH), adriamycin + cyclophosphamide sequential paclitaxel (AC-T), adriamycin + cyclophosphamide combined with paclitaxel (TAC), paclitaxel + carboplatin + trastuzumab (TCbH), and paclitaxel + cyclophosphamide + trastuzumab (TCH). Hormone receptor-positive cases received adjuvant endocrine therapy with the ER antagonist (TAM) tamoxifen in premenopausal patients and aromatase inhibitors, including letrozole, anastrozole, and exemestane, in postmenopausal patients (9). The anti-HER2 treatment of choice was trastuzumab (10). Radiation therapy was selected for some patients with tumors larger than 5 cm or 2–5 cm in maximum diameter and associated with more than 2–3 lymph node metastases (11). The disease-free survival (DFS) and overall survival (OS) of the two groups were compared over 5 years. All statistical data were analyzed in detail using SPSS 25.0 statistical software. The mean ± standard deviation was used to express the measurement data, and the count data was expressed as rate (%), and the χ2 test was selected. Univariate analysis was performed by the chi-square test; the Kaplan–Meier method was used to compare the survival data of patients in the two groups, and Cox regression was applied for multifactorial analysis of prognosis, with P < 0.05 indicating a statistically significant difference.

## 3 Results

### 3.1 Clinicopathological features of the 135 breast cancer patients

The triple-positive and HER2-positive groups showed no differences in age, menstrual status, tumor size, number of lymph node metastases, TNM stage, and Ki-67 index (P > 0.05). All 135 breast cancer patients in both groups underwent modified radical breast cancer surgery, with no significant intergroup difference in the choice of targeted therapy or radiotherapy after surgery (P > 0.05). Assessments of the chemotherapy regimen showed that 34 patients (56.7%) in the triple-positive group received AC-TH; 10 (16.7%) received AC-T; 6 (10%) received TAC; 2 (3.3%) received TCbH; and 8 (13.3%) received TCH; in the HER2-positive group, 25 patients (33.3%) received AC-TH; 20 (26.7%) received AC-T; 8 (10.7%) received TAC; 12 (16%) received TCbH; and 10 (13.3%) received TCH. The composition ratio of the choice of chemotherapy regimens differed significantly between the two groups (P = 0.029) (**Table 1**).

**Table 1:** Clinicopathological features of 135 breast cancer patients.

Variable	Group		Total	χ²	P
	Triple-positive	HER2-positive			
**Number**	**60**	**75**	**135**		
**Age (years, Mean ± SD)**	**49.42 ± 8.46**	**48.79 ± 10.66**			
Age (%)					
**<50 years**	**29 (48.3)**	**41 (54.7)**	**70 (51.9)**	**0.536**	**0.464**
**≥50 years**	**31 (51.7)**	**34 (45.3)**	**65 (48.1)**		
Menstrual status (%)					
**Non-menopausal**	**30 (50.0)**	**41 (54.7)**	**71 (52.6)**	**0.291**	**0.589**
**Menopausal**	**30 (50.0)**	**34 (45.3)**	**64 (47.4)**		
Maximum tumor diameter (%)					
**≤20 mm**	**26 (43.3)**	**37 (49.3)**	**63 (46.7)**	**1.338**	**0.595**
**>20 mm and ≤50 mm**	**34 (57.7)**	**37 (49.3)**	**71 (52.6)**		
**>50 mm**	**0 (0)**	**1 (1.4)**	**1 (0.7)**		
Number of lymph node metastases (%)					
**0**	**24 (40)**	**32 (42.7)**	**56 (41.5)**	**0.448**	**0.959**
**1-3**	**23 (38.3)**	**26 (34.7)**	**49 (36.3)**		
**4-9**	**10 (16.7)**	**14 (18.6)**	**24 (17.8)**		
**>10**	**3 (5)**	**3 (4)**	**6 (4.4)**		
TNM Staging(%)					
**I**	**12 (20)**	**20 (26.7)**	**32 (23.7)**	**1.199**	**0.576**
**II**	**35 (58.3)**	**37 (49.3)**	**72 (53.3)**		
**III**	**13 (21.7)**	**18 (24)**	**31 (23)**		
Ki-67 status (%)					
**<50%**	**49 (81.7)**	**51 (68)**	**100 (74.1)**	**3.242**	**0.072**
**≥50%**	**11 (18.3)**	**24 (32)**	**35 (25.9)**		
Endocrine therapy ≥ 5 years (%)					
**Yes**	**56 (93.3)**	**0 (0)**	**56 (41.5)**	**-**	**<0.001**
**No**	**4 (6.7)**	**75 (100)**	**79 (58.5)**		
Radiation therapy (%)					
**Yes**	**37 (61.7)**	**41 (54.7)**	**78 (57.8)**	**0.670**	**0.413**
**No**	**23 (38.3)**	**34 (45.3)**	**57 (42.2)**		
Targeted therapy (%)					
**Yes**	**44 (73.3)**	**47 (62.7)**	**91 (67.4)**	**1.726**	**0.189**
**No**	**16 (26.7)**	**28 (37.3)**	**44 (32.6)**		
Chemotherapy regimens (%)					
**AC-TH**	**34 (56.7)**	**25 (33.3)**	**59 (43.7)**	**10.824**	0.029*
**AC-T**	**10 (16.7)**	**20 (26.7)**	**30 (22.2)**		
**TAC**	**6 (10)**	**8 (10.7)**	**14 (10.4)**		
**TCbH**	**2 (3.3)**	**12 (16)**	**14 (10.4)**		
**TCH**	**8 (13.3)**	**10 (13.3)**	**18 (13.3)**		

AC-TH, adriamycin + cyclophosphamide sequential paclitaxel + trastuzumab; AC-T adriamycin + cyclophosphamide sequential paclitaxel; TAC, adriamycin + cyclophosphamide combined with paclitaxel; TCbH, paclitaxel + carboplatin + trastuzumab; TCH, paclitaxel + cyclophosphamide + trastuzumab.

### 3.2 Recurrence, metastasis, and survival of patients with triple-positive breast cancer and HER2-positive breast cancer

**Figure 1:**
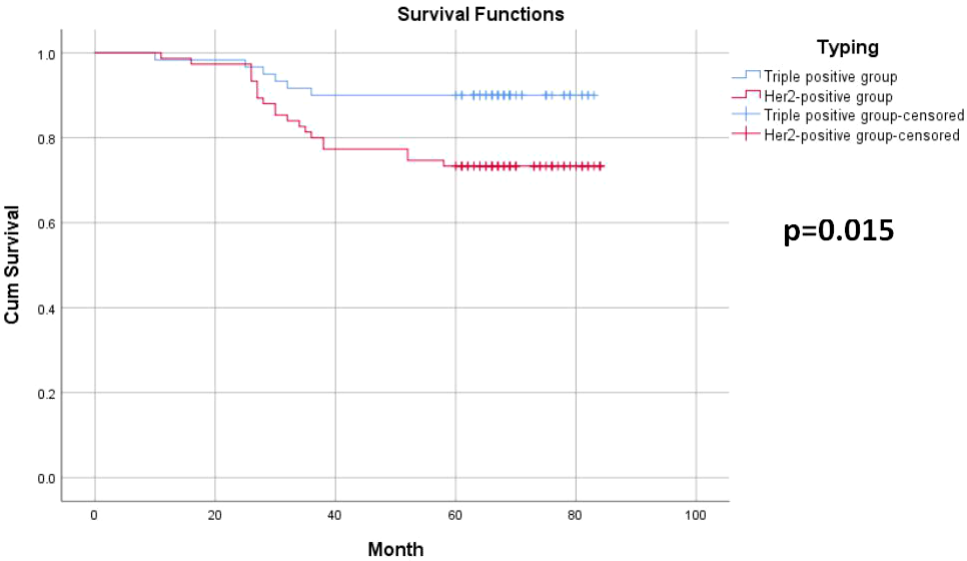
5-year disease-free survival survival curves for the two groups of breast cancer patients.

**Figure 2:**
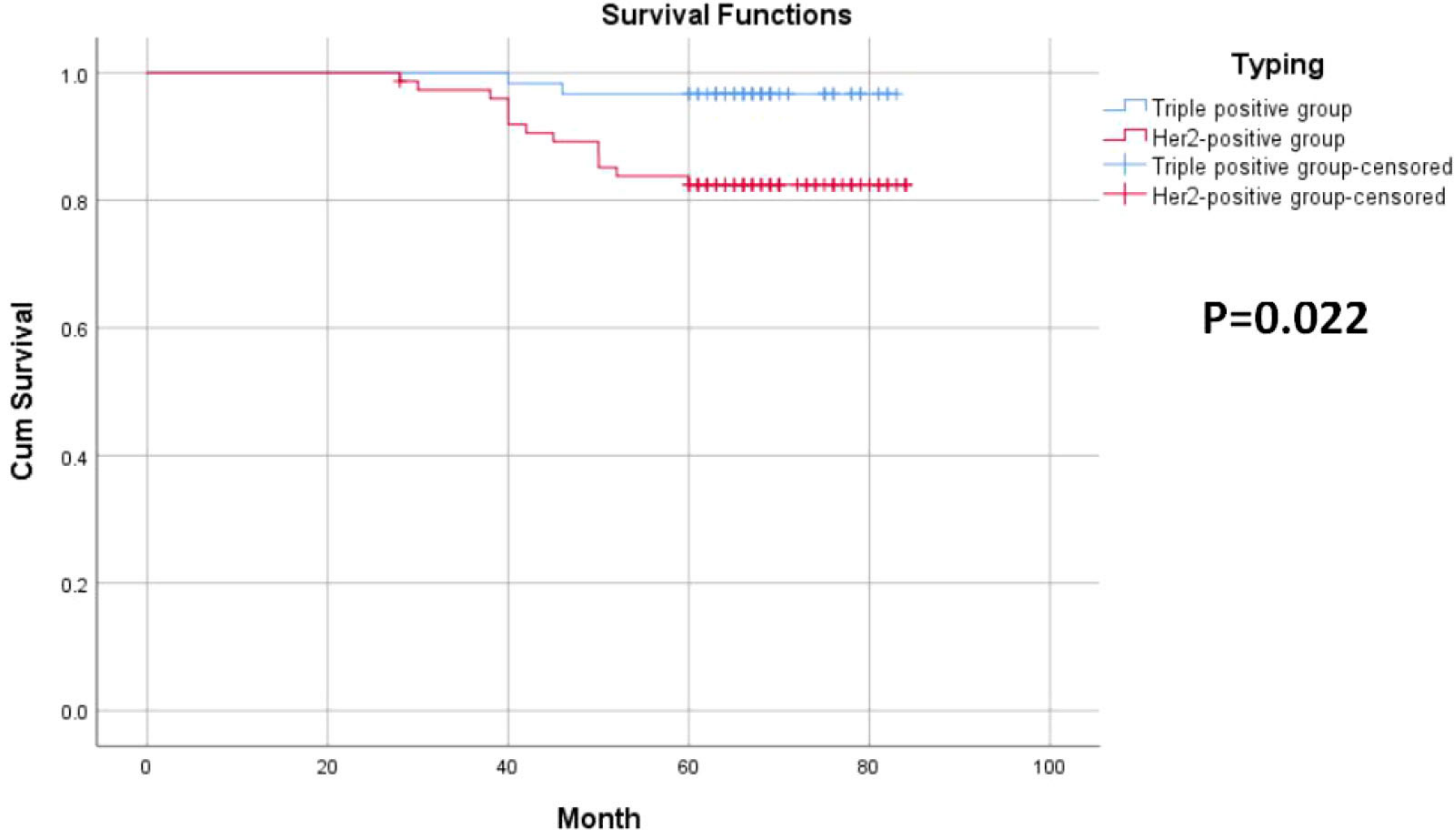
5-Year overall survival curves for the two groups of breast cancer patients.

Among the 135 breast cancer patients enrolled, 26 developed recurrence and metastasis, including six patients in the triple-positive group and 20 in the HER2-positive group. 15 patients died from malignant tumors, including two in the triple-positive group and 13 in the HER2-positive group. The 5-year OS rates of breast cancer patients in the two groups were 96.7% and 82.7%, respectively, and the 5-year DFS rates of patients in the two groups were 90% and 73.3%, respectively. The 5-year DFS and OS rates in the triple-positive group were significantly better than those in the HER2-positive group (P = 0.015, P = 0.022) (**Tables 2**, **3**). Survival analysis was performed using the Kaplan–Meier method in the two groups, and the results were shown in **Figures 1**, **2**.

**Table 2:** Recurrence and metastasis of triple-positive breast cancer and HER2-positive breast cancer.

Group	Recurrence and metastasis (%)		Total	5-year DFS(%)	χ²	P
	Yes	No				
**Triple-positive** **HER2-positive**	**6 (10)**	**54 (90)**	**60**	**90**	**5.954**	**0.015**
	**20 (26.7)**	**55 (73.3)**	**75**	**73.3**		

DFS, disease-free survival.

**Table 3:** Survival of triple-positive breast cancer and HER2-positive breast cancer.

Group	Death(%)		Total	5-year OS(%)	χ²	P
	Yes	No				
**Triple-positive** **HER2-positive**	**2 (3.3)**	**58 (96.7)**	**60**	**96.7**	**5.273**	**0.022**
	**13(17.3)**	**62 (82.7)**	**75**	**82.7**		

OS, overall survival.

### 3.3 Recurrence and metastasis of triple-positive breast cancer and HER2-positive breast cancer using different chemotherapy+targeted therapy

Among the 60 patients with triple-positive breast cancers, 34 were treated with AC-TH, of which two developed metastasis; 10 patients were treated with AC-T, of which three developed metastasis; six patients were treated with TAC, of which one developed metastasis; and two patients were treated with TCbH and eight patients were treated with TCH, of which none developed metastasis. The 5-year DFS rates did not differ significantly among multiple groups (P = 0.148), but the TCH and TCbH regimens yielded the longest DFS, while the AC-T regimen yielded the shortest DFS. Among the 75 patients with HER2-positive breast cancer, 25 received AC-TH, of which five developed metastasis; 20 received AC-T, of which 11 developed metastasis; eight received the TAC regimen, of which two developed metastasis; 12 received TCbH, of which two developed metastasis; and 10 received TCH, with no cases of metastasis. The 5-year DFS rates were significantly different across multiple cohorts (p = 0.011), and the AC-T regimen yielded the shortest DFS while the TAC and TCH regimens yielded the longest DFS (**Table 4**).

**Table 4:** Recurrence and metastasis in the triple-positive and HER2-positive groups using different chemotherapy regimens.

Group	Recurrence and metastasis (%)		Total	5-year DFS(%)	χ²	P
	Yes	No				
**Triple-positive group**			**60**			
**AC-TH**	**2_a_(5.9)**	**32_a_(94.1)**	**34**	**94.1**	**5.621**	**0.148**
**AC-T**	**3_b_(30)**	**7_a_(70)**	**10**	**70**		
**TAC**	**1_a_(16.7)**	**5_a_(83.3)**	**6**	**83.3**		
**TCbH**	**0_a_(0)**	**2_a_(100)**	**2**	**100**		
**TCH**	**0_a_(0)**	**8_a_(100)**	**8**	**100**		
**HER2-positive group**			**75**			
**AC-TH**	**5_a_(20)**	**20_a_(80)**	**25**	**80**	**11.999**	**0.011**
**AC-T**	**11_b_(55)**	**9_a_(45)**	**20**	**45**		
**TAC**	**2_a_(25)**	**6_a_(75)**	**8**	**75**		
**TCbH**	**2_a_(16.7)**	**10_a_(83.3)**	**12**	**83.3**		
**TCH**	**0_b_(0)**	**10_a_(100)**	**10**	**100**		

AC-TH, adriamycin + cyclophosphamide sequential paclitaxel + trastuzumab; AC-T adriamycin + cyclophosphamide sequential paclitaxel; TAC, adriamycin + cyclophosphamide combined with paclitaxel; TCbH, paclitaxel + carboplatin + trastuzumab; TCH, paclitaxel + cyclophosphamide + trastuzumab;DFS, disease-free survival.

The subscript letters indicate a subset of chemotherapy regimen categories, and the column proportions for these categories are not significantly different from each other at the 0.05 level.

Among the 60 patients with triple-positive breast cancer, 44 patients were treated with targeted therapy and two patients showed recurrence or metastasis; 16 patients were not treated with targeted therapy and four of these patients showed recurrence or metastasis. Among the 75 patients with HER2-positive breast cancer, 47 patients received targeted therapy and seven of these patients showed recurrence and metastasis; 28 patients did not receive targeted therapy and 13 of these patients showed recurrence or metastasis. DFS was significantly better in the trastuzumab subgroup than in the non-trastuzumab subgroup in both groups (P = 0.038, P = 0.003) (**Table 5**).

**Table 5:** Recurrence and metastasis with targeted drugs in the triple-positive and HER2-positive groups.

Group	Recurrence and metastasis (%)		Total	5-year DFS(%)	χ²	P
	Yes	No				
**Triple-positive group**			**60**			
**Targeted therapy**	**2 (4.50)**	**42 (95.5)**	**44**	**95.5**	**-**	**0.038**
**No targeted therapy**	**4 (25)**	**12 (75)**	**16**	**75**		
**HER2-positive group**			**75**			
**Targeted therapy**	**7 (14.9)**	**40 (85.1)**	**47**	**85.1**	**8.923**	**0.003**
**No targeted therapy**	**13 (46.4)**	**15 7(53.6)**	**28**	**53.6**		

DFS, disease-free survival.

### 3.4 Survival of triple-positive breast cancer and HER2-positive breast cancer patients with different chemotherapy+targeted therapy regimens

The 5-year OS rate was higher in the triple-positive group, and only two patients receiving the AC-T regimen died. The 5-year OS rate was the highest in the HER2-positive patients receiving TCH and lowest in those receiving AC-T (**Table 6**). The 5-year OS rate was significantly better in the triple-positive and HER2-positive subgroups treated with targeted drugs than in the group that did not receive targeted drugs (**Table 7**).

**Table 6:** Survival with different chemotherapy regimens in the triple-positive and HER2-positive groups.

Group	Death (%)		Total	5-year OS (%)	χ²	P
	Yes	No				
**Triple-positive group**			**60**			
**AC-TH**	**0_a_(0)**	**34_a_(100)**	**34**	**100**	**7.434**	**0.077**
**AC-T**	**2_b_(20)**	**8_a_(80)**	**10**	**80**		
**TAC**	**0_a_(0)**	**6_a_(100)**	**6**	**100**		
**TCbH**	**0_a_(0)**	**2_a_(100)**	**2**	**100**		
**TCH**	**0_a_(0)**	**8_a_(100)**	**8**	**100**		
**HER2-positive group**			**75**			
**AC-TH**	**4_a_(16)**	**21_a_(84)**	**25**	**84**	**4.041**	**0.382**
**AC-T**	**6_a_(30)**	**14_a_(70)**	**20**	**70**		
**TAC**	**1_a_(12.5)**	**7_a_(87.5)**	**8**	**87.5**		
**TCbH**	**2_a_(16.7)**	**10_a_(83.3)**	**10**	**83.3**		
**TCH**	**0_a_(0)**	**10_a_(100)**	**10**	**100**		

AC-TH, adriamycin + cyclophosphamide sequential paclitaxel + trastuzumab; AC-T adriamycin + cyclophosphamide sequential paclitaxel; TAC, adriamycin + cyclophosphamide combined with paclitaxel; TCbH, paclitaxel + carboplatin + trastuzumab; TCH, paclitaxel + cyclophosphamide + trastuzumab;OS, overall survival.

The subscript letters indicate a subset of chemotherapy regimen categories, and the column proportions for these categories are not significantly different from each other at the 0.05 level.

**Table 7:** Survival with targeted drugs in the triple-positive and HER2-positive groups.

Group	Death (%)		Total	5-year OS (%)	χ²	P
	Yes	No				
**Triple-positive group**			**60**			
**Targeted therapy**	**0 (0)**	**44 (100)**	**44**	**100**	**—**	**0.068**
**No targeted therapy**	**2 (12.5)**	**14 (87.5)**	**16**	**87.5**		
**HER2-positive group**			**75**			
**Targeted therapy**	**6 (12.8)**	**41 (87.2)**	**47**	**87.2**	**1.873**	**0.176**
**No targeted therapy**	**7 (25)**	**21 (75)**	**28**	**75**		

OS, overall survival.

### 3.5 Multifactorial regression analysis

A multifactorial Cox risk model was constructed by grouping age, menstrual status, maximum tumor diameter, number of lymph node metastases, pathological staging, and Ki-67 findings. The results revealed that molecular staging was an independent factor (p = 0.012) affecting breast cancer prognosis (p = 0.005) and that patients in the HER2-positive group had a higher risk of recurrent metastasis than those in the triple-positive group (HR = 2.199; 95% CI, 1.296-8.266) (**Table 8**). The risk of death was higher in the HER2-positive group than in the triple-positive group (HR = 9.994; 95% CI, 2.019-49.465) (**Table 9**).

**Table 8:** Multifactorial Cox analysis of 5-year disease-free survival of the 135 breast cancer patients.

Variables	Group	B	P	HR	95.0% CI	
					Lower limit	Upper limit
**Age**		**0.037**	**0.277**	**1.038**	**.971**	**1.109**
**Menstrual status**	Non-menopausal*	**0**				
	**Menopausal**	**-0.185**	**0.797**	**0.831**	**.204**	**3.389**
**pT-staging**		**0.509**	**0.212**	**1.663**	**.748**	**3.699**
**pN-staging**		**0.731**	**0.000**	**2.078**	**1.386**	**3.116**
**Ki-67 status**	<50%*	**0**				
	**≥50%**	**0.788**	**0.069**	**3.273**	**0.942**	**5.134**
**Typing**	Triple-positive*	**0**				
	**HER2-positive**	**1.186**	**0.012**	**2.199**	**1.296**	**8.266**

*Control group.

**Table 9:** Multifactorial Cox analysis of the 5-year overall survival of 135 breast cancer patients.

Variables	Group	B	P	HR	95.0% CI	
					Lower limit	Upper limit
**Age**		**0.010**	**-0.816**	**1.010**	**0.930**	**1.097**
**Menstrual status**	Non-menopausal*	**0**				
	**Menopausal**	**-2.313**	**0.040**	**0.099**	**0.011**	**0.902**
**pT-staging**		**1.592**	**0.025**	**4.915**	**1.219**	**19.819**
**pN-staging**		**1.342**	**.000**	**3.827**	**2.084**	**7.030**
**Ki-67 status**	<50%*	**0**				
	**≥50%**	**1.567**	**0.015**	**4.791**	**1.352**	**16.977**
**Typing**	Triple-positive*	**0**				
	**HER2-positive**	**2.302**	**0.005**	**9.994**	**2.019**	**49.465**

*Control group.

## 4 Discussion

Thanks to medical advances, both early diagnosis and optimal treatment can increase the survival duration of patients with breast cancer. Regardless of the molecular staging of early breast cancer, surgery remains one of the most effective treatment modalities for these patients (12). However, the possibility of postoperative recurrence cannot be ruled out. Surgery can be followed by chemotherapy, radiotherapy, and targeted endocrine therapy to reduce tumor metastasis and recurrence, thus prolonging survival (13). Moreover, postoperative immunohistochemical testing can clarify the molecular type of breast cancer, providing guidance for treatment and prognosis. ER, PR, HER2, and Ki-67 are commonly evaluated for the diagnosis and treatment of breast cancer. These indicators can reflect the growth, invasion, and recurrence of tumor cells, and also facilitate the development of personalized treatment plans for clinical purposes. Immunohistochemistry results guide TNM staging of breast cancer. This paper addresses the status of hormone receptors (HR+ versus HR-) to study the prognosis of two types of HER2-positive breast cancers, which are highly aggressive and have a high recurrence rate and high risk of metastasis, accounting for approximately 20%-30% of all breast cancers (14). Approximately 50% of HER2-positive breast cancers are HR-positive, i.e. triple-positive breast cancers, while the proportion of HR-negative breast cancers is approximately 30%.ER-positive PR-negative accounts for 15-20%, the existence of ER-negative PR-positive is still controversial.

In this study, Cox regression analysis showed that tumor size and the number of lymph node metastases were independent prognostic factors affecting patients’ 5-year OS and DFS (P < 0.05). The maximum tumor diameter and the number of lymph node metastases as well as the presence of distant metastases constitute the TNM stage: the larger the tumor diameter, greater was the extent of lymph node metastases and the more advanced the TNM stage, indicating a poor prognosis. Moreover, these aspects usually affected the choice of treatment. Larger tumors are less favorable for patients who wish to preserve their breasts and may often require patients to receive chemotherapy prior to surgery to shrink the tumor before surgery. In addition, one study (15) found that patients with HR-negative/HER2-positive tumors had more aggressive clinical features, including tumor stages III-IV, T stages 2-4, N stages 1-3, and M stage 1 including brain, liver, and lung metastases, clinical research revealed that BC metastasized mostly to the lung, bone, and liver *via* circulatory system (16); In contrast, triple positivity was associated with milder tumor behavior. These results suggest that triple-positive tumors are an independent biological subtype.

The study conducted by Prof. Zhimin Shao and his team pooled five study cohorts from public databases as well as the Department of Breast Surgery at the Affiliated Cancer Hospital of Fudan University (17). Their clinical and multi-omics data analysis revealed that in comparison with HR-/HER2+ breast cancer, patients with triple-positive breast cancer generally have smaller tumor diameters, relatively lower lymph node metastasis rates, and better prognoses. The 5-year OS rates of patients with HR+/HER2+ versus HR-/HER2+ breast cancer in our study were 96.7% and 82.7%, respectively, and the 5-year DFS rates of patients in the two groups were 90% and 73.3%, respectively. The findings for the triple-positive group were significantly better than those for the HER2-positive group, and the differences between the two groups were statistically significant (P = 0.022, P = 0.015). However, the prognosis of triple-positive and HER2-positive breast cancer is controversial. HER2 is the driver gene of breast cancer, and patients with HER2-positive breast cancer have shorter survival and poorer prognosis, while the BIG1-98 study showed that the prognosis of patients with triple-positive breast cancer remained poor even after 5-10 years of endocrine therapy (18). Positive or high levels of HR have been shown to reduce the sensitivity of anti-HER2 therapy, resulting in reduced benefit for this group of patients, and HER2 overexpression also reduces the sensitivity of endocrine therapy (19). In triple-positive breast cancer patients, the ER and HER2 pathways interact through complex intracellular signaling mechanisms to promote tumor growth, but because of the mutual crosstalk between the HER2 signaling pathway and HR channels, interventions in one pathway may affect the other pathway to varying degrees; for example, HER2 overexpression-mediated activation of PI3K-AKT-mTOR and MAPK pathway activation downregulates ER expression, leading to resistance to endocrine therapies, while ER activates EGFR, HER2, IGFR1, and other signaling pathways by binding to related ligands, inhibiting apoptosis and thus leading to tumorigenesis and progression (20).

Over the last 20 years, HER2-targeted therapy has led to a significant improvement in the survival of HER2-positive patients (21). The results of many clinical trials have shown that patients can benefit from single, dual-target, or T-DM1 therapy in neoadjuvant, adjuvant, and late-stage settings, regardless of HR status. HR+/HER2+ breast cancer remains the predominant type of breast cancer with the HER2 signaling pathway, requiring anti-HER2+ therapy followed by a combination of endocrine therapies depending on HR expression. In this study, the 5-year DFS rates were 95.5% and 75% for the triple-positive group with and without targeted agents, respectively, and the corresponding values for the HER2-positive group were 85.1% and 53.6%, respectively, with statistically significant differences (P = 0.038, P = 0.003). Similarly, the 5-year OS rates were 100% and 87.5% for the triple-positive group with and without targeted agents, respectively, and the corresponding values for the HER2-positive group were 87.2% and 75%, respectively. Thus, the data clearly showed that the prognosis of triple-positive breast cancer is better than that of HER2-overexpressing breast cancer regardless of the application of targeted therapy. In addition, the prognoses of both breast cancer groups with targeted drugs were also significantly better than those of the groups without targeted drugs. The clinical status of trastuzumab as a first-line agent was established by the H0648g/M77001 study. The results of the H0684g study (22) showed that the OS was 20.3 months in the chemotherapy alone group and increased to 25.1 months in the trastuzumab plus chemotherapy group (P = 0.046), and the difference was statistically significant; the results of the M77001 study (23) also showed that the OS was 31.2 months in the trastuzumab combined with docetaxel group and 22.7 months in the docetaxel alone group (P = 0.0325). In a phase III HERA trial (24) that included 5102 HER2-positive early-stage breast cancers, the group receiving trastuzumab showed a significant reduction in the risk of disease-free survival events and death in comparison with the observation group that did not receive it, with a 24% relative reduction in the risk of DFS events and 26% relative reduction in the risk of death after 1 year of trastuzumab use by patients. Moreover, in the N9831/B31 study (25), which included 4046 patients with HER2-positive breast cancer, the 10-year DFS and OS rates were 73.7% and 84% in the treatment group with AC-T, and 62.2% and 75.2% in the treatment group without AC-T, respectively. Thus, the significance of anti-HER2 therapy for HER2-positive breast cancer is easy to determine.

Nevertheless, single-target adjuvant therapy is associated with 25% probability of recurrence, especially in cases with positive lymph nodes. Therefore, assessment of the risk of recurrence for such patients, e.g. by evaluation of lymph node status and HR status, can be considered to be the most appropriate treatment strategy. For T1 early-stage tumors, a step-down treatment with streamlined chemotherapy can be chosen, and TCH and weekly TH regimens can also meet the treatment needs of the patients. However, an elevated risk of recurrence may necessitate AC-T in combination with a single- or even dual-target adjuvant therapy (26). The APHINITY study (27) found that dual-target adjuvant therapy further reduced the risk of recurrence by 24%, with a significant benefit for those with lymph node metastases and a reduction of 28% in the risk of recurrence, while the results over the next 6 years of follow-up showed that dual targeting not only reduced the risk of recurrence in HR-/HER2+ patients, but HR+ patients with late recurrence also achieved a statistically significant improvement in survival at a median follow-up period of 74.1 months. The ALTTO study (28) found that management of HER2-positive breast cancer can be divided into two different treatment strategies and follow-up modalities specifically based on HR status and natural disease duration. Thus, when ER/PR is >50%, there is no significant difference in the efficacy of targeted therapy combined with or without chemotherapy (29). Therefore, the study proposed that the treatment regimen of some triple-positive breast cancer patients involved overtreatment. A US study included 6234 patients with triple-positive breast cancer and found that 60% of patients were willing to receive endocrine therapy rather than chemotherapy and had significantly higher 5-year survival rates compared to chemotherapy (from 39.8% to 47.5%) (30). Several studies such as ALTERNATIVE have shown that HR+/HER2+ patients showing good results with depot chemotherapy and low toxicity after endocrine therapy, suggesting that such therapeutic approaches can be the first choice of treatment for those who are sensitive to endocrine therapy as well as those who cannot tolerate chemotherapy. The combination of endocrine therapy with targeted when HRs are highly expressed is a better treatment option than combining with chemotherapy. CDK4/6 is a key regulator of the cell cycle, and inhibition of its pathway is slowly becoming a possible therapeutic strategy for this type of breast cancer, with the opportunity to achieve blockade at the intersection of the ER and HER2 dual pathways (31). The NA-PHER2 study (32) suggested this idea, and reported a 27% pCR rate and 97% efficacy of neoadjuvant therapy with a dual-targeted combination of CDK4/6 inhibitors (paboxetine) with fulvestrant in this group of patients. Therefore, should triple-positive breast cancer still continue with the same one-size-fits-all targeted combination chemotherapy regimen as HER2-positive breast cancer? More data from large clinical studies are still needed to conclusively answer this query. We are still at the stage of categorizing and treating based on the risk of recurrence as well as molecular typing to select adjuvant treatment options. However, in the current era of individualized precision medicine, biomarkers are also needed to precisely screen patients for therapeutic benefit and explore their value in HER2 positive breast cancer.

The present study was a single-center retrospective study, which may have resulted in some bias in data collection, and the small sample size, the surgical treatment of included patients who all underwent modified radical breast cancer, and the short follow-up period may have influenced the study results, so a clinical study with a larger sample size is needed to improve the objectivity of the results.

## 5 Conclusion

The 5-year DFS and OS of triple-positive breast cancer are significantly better than Her2 overexpression breast cancer, and the prognosis is also significantly different after using the same chemotherapy regimen. Therefore, We should fully evaluate the risk of recurrence of breast cancer patients, such as hormone receptor status, HER2 expression, lymph node metastasis, etc., and then choose the appropriate treatment plan to bring the greatest benefit to patients.

## Data availability statement

The datasets presented in this article are not readily available because the original contributions presented in the study are included in the article/supplementary material. Further inquiries can be directed to the corresponding authors. Requests to access the datasets should be directed to XS sddxsxc@126.com.

## Ethics statement

The studies involving human participants were reviewed and approved by Shandong Provincial Hospital ethics committee Affiliated to Shandong First Medical University. The patients/participants provided their written informed consent to participate in this study.

## Author contributions

XS and XT conceived the study. All authors collected data. DM, QY and XC analyzed data. KY followed up with data. DM wrote the manuscript. All authors contributed to the article and approved the submitted version.
